# Distinct BCR repertoires elicited by SARS-CoV-2 RBD and S vaccinations in mice

**DOI:** 10.1038/s41421-021-00331-9

**Published:** 2021-10-07

**Authors:** Siyu Tian, Kai Ji, Meng Wang, Fengze Wang, Hao Wang, Weijin Huang, Qingrui Huang, Jinghua Yan

**Affiliations:** 1 CAS Key Laboratory of Microbial Physiological and Metabolic Engineering, Institute of Microbiology, Chinese Academy of Sciences, Beijing, China; 2 University of Chinese Academy of Sciences, Beijing, China; 3 National Division of HIV/AIDS and Sex-transmitted Virus Vaccine, National Institutes for Food and Drug Control (NIFDC) and WHO Collaborating Center for Standardization and Evaluation of Biologicals, Beijing, China

**Keywords:** Immunology, Molecular biology

Dear Editor,

The ongoing coronavirus disease 2019 (COVID-19) pandemic, caused by severe acute respiratory syndrome coronavirus 2 (SARS-CoV-2), has resulted in serious public health and economic crisis worldwide^1^. Among the approved vaccines and vaccine candidates in clinical evaluation, except inactivated virus vaccines, the others make use of SARS-CoV-2 receptor binding domain (RBD) or spike protein (S) as vaccine immunogen^2–8^. However, the precise nature comparison of antibody repertoires induced by RBD and S vaccinations, especially through vaccines developed by the same vaccine platform, has been little investigated. Here we dissected mouse memory B cell receptor (BCR) V(D)J profile following SARS-CoV-2 RBD and S mRNA vaccinations by high-throughput single-cell sequencing (scRNA-seq) to yield important insights into antibody responses to different SARS-CoV-2 antigens.

We initially designed two mRNA constructs expressing RBD and the 6P variant of the full-length S protein (S-6P)^9^ (Supplementary Fig. S1a). The S-6P variant was constructed by introducing four additional beneficial proline substitutions (F817P, A892P, A899P, and A942P) with an aim to increase protein yield and prefusion stability on the basis of the 2P variant utilized by mRNA-1273 and BNT162b2 vaccines^9^. mRNA transfection of HEK293T cells confirmed robust protein expression in culture supernatants for the RBD construct and in cell lysates for the S construct (Supplementary Fig. S1b). Mice were immunized with two doses of 15 μg RBD or S mRNA vaccines or placebo at a 4-week interval. At 8 weeks post initial vaccination, the RBD vaccine elicited comparable S-specific immunoglobulin G levels to the S vaccine with both titers reaching up to 10^6^ (Supplementary Fig. S2a), whereas the neutralizing antibody titer induced by S vaccinations was significantly higher than that by RBD vaccinations (Supplementary Fig. S2b), suggesting a potential correlation of recognized epitope profile by the antibody repertoire to its neutralizing activity. Next, we assayed neutralizing activity of mouse vaccinated sera against currently emerging SARS-CoV-2 variants. The RBD- and S-elicited sera exhibited convergent trends for decrease or increase in neutralization activity against all 32 pseudoviruses in the study, and only the G476S and S477N mutations led to a 1.2–1.8-fold decrease of the neutralization activity for both RBD- and S-vaccinated sera (Supplementary Fig. S2c–e).

At 8 weeks following initial immunization, SARS-CoV-2-specific germinal center memory B cells (B_GC_) from the lymph nodes were sorted by flow cytometry (Supplementary Fig. S3) and then placed into the 10× Chromium (10× Genomics) for high-throughput single-cell V(D)J sequencing to obtain accurately paired full-length variable regions (Supplementary Fig. S4). As a result, from the RBD group, 3615 variable regions for heavy chain (VH) and 4163 variable regions for light chain (VL) were obtained with 3048 in pairs consisting of 1611 clones; from the S group, 1505 VH and 1684 VL were obtained with 1227 in pairs consisting of 762 clones (Fig. 1a). The V gene germline usage by the RBD and S groups presented distinct spectrums for both VH and VL (Fig. 1a and Supplementary Table S1). In detail, the most frequent germline presented by SARS-CoV-2-specific B_GC_ cells in the RBD group, VH9-3 (15.6%) and KV5-45 (13.5%), only accounts for 3.8% and 0.2% in the S group, respectively (Fig. 1a and Supplementary Table S1). To further analyze germline usage in SARS-CoV-2-specific B_GC_ cells for the RBD and S groups, we also figured out a whole VH and VL pairing (VH:VL) landscape for the top 20 used germlines of VH and KV for each group. Impressively, the RBD group predominantly utilized one germline combination, VH9-3:KV5-45, and its frequency was as high as 14.6% (Fig. 1b). In contrast, the most dominant germline combination used by the S group was VH5-9-1:KV13-84, and its frequency was < 5%, and the combined germlines used a larger germline pool for both VH and VL with more dispersed gene frequencies (Fig. 1b).

**Fig. 1:**
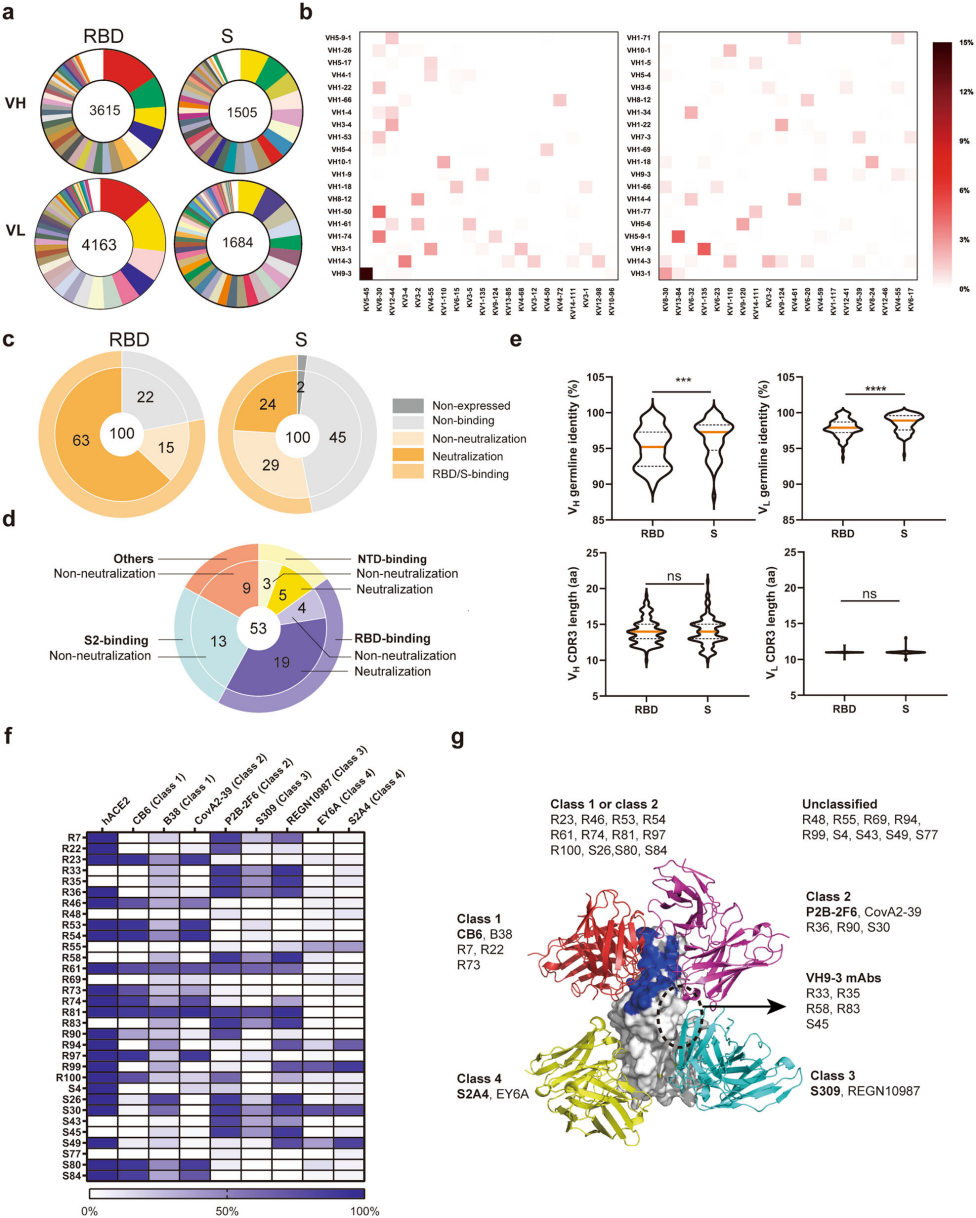
Distinct BCR repertoires elicited by SARS-CoV-2 RBD and S vaccinations in mice. SARS-CoV-2 antigen (mixture of RBD and S proteins) positive B_GC_ cells in lymph nodes from vaccinated mice were sorted by flow cytometry and then placed into the 10× Chromium (10× Genomics) for high-throughput single-cell V(D)J sequencing. **a** Pie charts show clonal expansion of SARS-CoV-2-specific B cells in the germinal center for IGVH (upper panel) or IGVL (bottom panel). Colored slices are proportional to the number of clonal relatives. See also related Supplementary Table S1. **b** Paired V gene use in B cell repertoire induced by SARS-CoV-2 vaccines based on RBD (left) or S (right). **c** Proportions of expressed, binding, and neutralizing antibodies in the top 100 frequent recovered mAbs in antibody repertoire. See also related Supplementary Fig. S5. **d** Proportions of the indicated antigenic site-directed, non-neutralizing and neutralizing antibodies in 53 recovered S-reactive mAbs from the S group. See also related Supplementary Fig. S5. **e** The gene germline identity and the CDR3 length of VH and VL of all synthesized mAbs from RBD and S groups is shown on the top and bottom, respectively. **f** Competition heatmap of representative RBD-reactive neutralizing mAbs from RBD and S repertoires with hACE2 and reference mAbs CB6 (class 1), B38 (class 1), CovA2-39 (class 2), P2B-2F6 (class 2), S309 (class 3), REGN10987 (class 3), EY6A (class 4), and S2A4 (class 4). **g** Structural depiction of reference mAbs CB6 (red, PDB 7C01), P2B-2F6 (magenta, PDB 7BWJ), S309 (cyan, PDB 6WPS), and S2A4 (yellow, PDB 7JVC) RBD-binding epitopes. RBD residues involved in hACE2 binding was colored as blue. Representative RBD-reactive neutralizing mAbs were classified based on their competition with hACE2 and reference mAbs binding to RBD. A dotted circle indicates a deduced possible epitope of VH9-3 mAbs on RBD that locates between P2B-2F6 and S309 epitopes and to some extent overlaps with both epitopes but does not overlap with hACE2-binding site. For **c**, **d**, **f**, data are one representative result of two or three independent experiments. For **d**, comparison was performed by Student’s *t-*test (unpaired, two-tailed).

To further characterize the antibodies elicited by RBD and S mRNA vaccines, we recovered 100 mouse monoclonal antibodies (mAbs) per group, representing the top 100 frequent clones, for frequency 40.26% of the RBD group and 41.81% of the S group. The results demonstrated that, for the RBD group, all 100 mAbs were expressed, and among them, 78 (78%) bound RBD with 63 (63%) showing neutralizing activities (IC_50–100%_ < 6 μg/mL) and 42 (42%) exhibiting the most potent neutralizing activity (IC_50–100%_ < 0.1 μg/mL); for the S group, 98 (98%) mAbs were expressed, and among them, 53 (53%) bound to S trimer protein, which included 24 (24%) scored as neutralizing positive and 10 (10%) neutralizing SARS-CoV-2 with IC_50–100%_ < 0.1 μg/mL (Fig. 1c and Supplementary Fig. S5). Of the 53 S-reactive mAbs in the S group, 23 (43%), 8 (15%), and 13 (25%) targeted RBD, N-terminal domain (NTD), and S2 subunit, respectively, and 9 (17%) belonged to “others” (non-RBD, non-NTD or non-S2 region in S trimer) (Fig. 1d and Supplementary Fig. S5). Nineteen (36%) RBD-targeting and 5 (9%) NTD-targeting mAbs showed neutralizing activity, whereas none of the S2-directed mAbs and those in “others” were detected positive for neutralization (Fig. 1d and Supplementary Fig. S5). All five NTD-targeting neutralizing antibodies (S2, S3, S8, S71, and S76) evolved from distinct germlines, whereas 11 of the 19 RBD-targeting neutralizing antibodies from similar VH1-5, VH1-22, or VH1-26 germlines (Supplementary Figs. S5 and S6). Moreover, mixture of S8 (an NTD-targeting mAb) and S45 or S80 (RBD-targeting mAbs) showed enhanced neutralization of live SARS-CoV-2 virus in a synergic manner (Supplementary Fig. S7). Next, we tested all RBD-directed mAbs for their ability of blocking the binding of RBD to hACE2 using an Octet-based binding assay or a fluorescence-activated cell sorter-based assay. There were 35 and 13 potent neutralizing mAbs from the RBD and S groups, respectively, which can completely block the binding between hACE2 and RBD (Supplementary Figs. S5 and S8). All VH9-3 mAbs from both the RBD and S groups with the most potent neutralizing activities showed no or weak blocking of hACE2 binding to RBD (Supplementary Figs. S5 and S8). Notably, the V gene germline identity of synthesized mAbs from the RBD group was significantly lower than those induced by the S vaccinations (Fig. 1e), potentially indicating a more advanced affinity maturation.

Structural comparisons allowed RBD-targeted antibodies to be broadly classified into four main classes^10^. Next, we performed a competition-binding assay using hACE2, CB6 (class 1), B38 (class 1), CovA2-39 (class 2), P2B-2F6 (class 2), S309 (class 3), REGN10987 (class 3), EY6A (class 4), and S2A4 (class 4) as reference detecting mAbs for 32 RBD-directed representative neutralizing mAbs (selected based on their VDJ sequences) from RBD and S repertoires to gain insight into their epitopes. Most of mAbs from both RBD- and S-elicited antibody repertoires belonged to class 1 or class 2, and no typical class 3 or class 4 mAb that did not block hACE2 but blocked the corresponding reference detecting mAbs such as S309 or S2A4 was identified (Fig. 1f, g and Supplementary Fig. S9). Of note, all five representative VH9-3 mAbs (four from the RBD group and one from the S group) failed to block hACE2 but largely and partially competed with P2B-2F6 (class 2) and S309 (class 3), respectively (Fig. 1f, g and Supplementary Figs. S8 and S9). Thus, we deduced that those mAbs targeted epitopes that may locate between P2B-2F6 and S309 epitope, partially overlap with both, but do not overlap with the hACE2-binding region (Fig. 1g). Moreover, there were five and four mAbs unclassified from the RBD and S group, respectively, because of either their non-competition with all reference detecting mAbs or a limited epitope information provided by the binding competition assay (Fig. 1f, g and Supplementary Fig. S9).

In summary, we dissected mouse memory B cell BCR profile following SARS-CoV-2 RBD and S mRNA vaccinations by high-throughput scRNA-seq. The RBD-elicited antibody repertoire demonstrated a strong enrichment in a gene germline combination usage of VH9-3:KV5-45. In contrast, for the S-elicited antibody repertoire, there was no particularly favored VH and VL germline combination usage identified, and antibodies were diverse in both gene germline usage and epitope recognition of the S protein. In the S group, both RBD- and NTD-directed neutralizing mAbs were screened out, whereas all S2-directed mAbs and those in “others” were scored as neutralizing negative. In addition, all recovered VH9-3 mAbs from both RBD and S repertoires exhibited the most potent neutralizing activity with weak or no blocking of hACE2 binding to RBD. Competition binding assays indicated that their epitopes possibly located between P2B-2F6 (class 2) and S309 (class 3) epitopes, partially overlapped with both, but non-overlapped with the hACE2-engaging region.

Genetic variants of SARS-CoV-2 have been emerging and circulating around the world throughout the COVID-19 pandemic. Some variants of concern (VOCs) such as B.1.351, P.1, and B.1.617.2 lineages have been reported to exhibit compromised efficacy of mAbs and vaccines^11^. Our study showed that, in contrast to RBD, the S immunogen elicited diverse neutralizing mAbs that targeted not only RBD but also NTD. Those diverse mAbs have potential to exert potent neutralizing activity against SARS-CoV-2 through synergistic effect and are generally beneficial to combat against virus variants. However, in contrast to neutralizing RBD-directed antibodies that recognize multiple non-overlapping epitopes, potent NTD-directed neutralizing antibodies appear to target a single supersite^12^. Moreover, a lot of SARS-CoV-2 variants, including the VOC B.1.1.7, B.1.351, P.1, and B.1.617.2 lineages, harbor frequent mutations within the NTD supersite and lead to neutralizing activity loss of those NTD-directed mAbs^11^. Thus, next-generation vaccine design needs to take all those above factors into consideration.

## Supplementary information

Supplementary Information
