# Supplementary material for: Distinct BCR repertoires elicited by SARS-CoV-2 RBD and S vaccinations in mice
Source: Cell Discov. 2021 Oct 7;7:91. doi: 10.1038/s41421-021-00331-9 (PMC8495183; doi:10.1038/s41421-021-00331-9)
Supplement: Supplementary file 1 — Supplementary Information [file 41421_2021_331_MOESM1_ESM.pdf]

Distinct BCR repertoires elicited by SARS-CoV-2 RBD and S vaccinations in mice

Authors: Siyu Tian[1,2*], Kai Ji[1,2*], Meng Wang[3,*], Fengze Wang[1,2], Hao Wang[1,2], Weijin Huang[3†], Qingrui Huang[1†],Jinghua Yan[1,2†].

Affiliations:

[1]CAS Key Laboratory of Microbial Physiological and Metabolic Engineering, Institute of Microbiology, Chinese Academy of Sciences, Beijing 100101, China.

[2]University of Chinese Academy of Sciences, Beijing 101408, China.

[3] Division of HIV/AIDS and Sex-transmitted Virus Vaccine, National Institutes for Food and Drug Control (NIFDC) and WHO Collaborating Center for Standardization and Evaluation of Biologicals, Beijing, 102629, China.

[†]Correspondence to: huangweijin@nifdc.org.cn (Weijin Huang); huangqr@im.ac.cn (Qingrui Huang); yanjh@im.ac.cn (Jinghua Yan).

[*]These authors contributed equally to this work.

Materials and methods

Ethics statement

This study was carried out in strict accordance with the recommendations described in the Guide for the Care and Use of Laboratory Animals of the Institute of Microbiology, Chinese Academy of Sciences (IMCAS) Ethics Committee. All the animal experiments were reviewed and approved by the Committee on the Ethics of Animal Experiments of IMCAS.

Cells, viruses, and animals

HEK293T cells (ATCC CRL-3216) and Vero E6 cells (ATCC CRL-1586) were cultured at 37°C in Dulbecco's modified Eagle's medium (DMEM) supplemented with 10% fetal bovine serum (FBS). The SARS-CoV-2 strain hCoV-19/China/CAS-B001/2020 (GISAID ID: EPI_ISL_514256-7) used in this study was isolated by Dr. Yuhai Bi and was stored in our laboratory. Vero E6 cells were applied to the amplification and titration of the virus stocks. BALB/c mice were purchased from Beijing Vital River Animal Technology Co., Ltd (licensed by Charles River) and were housed and bred in the temperature-, humidity- and light cycle-controlled animal facility (20 ± 2°C; 50 ± 10%; light, 7:00–19:00; dark, 19:00–7:00) of specific-pathogen-free (SPF) mouse facilities in IMCAS. All experiments involving infectious SARS-CoV-2 were performed under Biosafety Level 3 facilities in IMCAS, which is approved for such use by of National Health Commission of China.

mRNA production

mRNA was produced using T7 RNA polymerase on linearized plasmids (synthesized by Genescript) encoding codon-optimized SARS-CoV-2 RBD glycoproteins (residues 319–541, accession number YP_009724390) or SARS-CoV-2 full-length S (the 6P variant) glycoproteins. The mRNA was transcribed to contain a 104 nucleotide-long poly(A) tail, and 1-

methylpseudourine-5′-triphosphate was used instead of UTP to generate modified nucleoside-containing mRNA. The mRNA was purified by overnight LiCl precipitation at -20°C, centrifuged at 18,800 ×g for 30 min at 4 °C to pellet, washed with 75% EtOH, centrifuged at 18,800 ×g for 1 min at 4 °C, and resuspended in RNase-free water. The purified mRNA was analyzed by agarose gel electrophoresis and stored frozen at -80°C until use.

mRNA transfection

Transfection of HEK293T cells was performed with TransIT-mRNA (Mirus Bio) according to the manufacturer's instructions. In brief, mRNA (0.5 μg) was combined with TransIT-mRNA regent (1 μl) and boost reagent (1 μl) in 50 μl of serum-free medium, and the complex was added to 2.5×10^5cells in 500μl complete medium. The supernatant was collected and concentrated, and cells were lysed on ice in NP-40 lysis buffer (GenStar) at 48 h after transfection.

Western blot

Whole-cell lysates and supernatants from cells transfected with RBD or S mRNA were assayed for expression by Western blotting. Samples were combined with loading buffer with dithiothreitol and separated by 10% SDS-PAGE. Transfer to a NC membrane was performed using a semi-dry apparatus (Ellard Instrumentation). The membrane was blocked with non-fat milk in TBS buffer containing 0.5% Tween-20. RBD and S protein was detected using serum from mice immunized with SARS-CoV-2 S protein (Sino Biological) for 1 h, followed by secondary goat anti-mouse IgG-HRP (Yeasen) for 1 h. The membrane was developed by SuperSignal West Pic chemiluminescent substrate (Thermo Fisher Scientific).

Lipid-nanoparticle encapsulation of mRNA

SARS-CoV-2 RBD- and S-encoded mRNA was encapsulated in LNPs by using a self-assembly process in which an aqueous solution of mRNA at pH=4.0 was rapidly mixed with a solution of

lipids dissolved in ethanol. LNPs used in this study contained an ionizable cationic lipid, phosphatidylcholine, cholesterol, and PEG-lipid at a ratio of 50:10:38.5:1.5 mol/mol and were encapsulated at an mRNA to lipid ratio of around 0.05 (wt/wt). Formulations were then diafiltrated against Phosphate Buffered Saline (PBS) and concentrated to desired concentrations, passed through a 0.22 um filter, and stored at 4 °C at a concentration of RNA of about 1 mg/ml.

Animal experiments

LNPs-encapsulated RBD and S mRNA was diluted with PBS. Female BALB/c mice aged six-eight weeks were inoculated intramuscularly (i.m.) with 15 µg RBD or S mRNA LNP formulations or poly(C) LNP formulations as placebo control, and boosted with the same dose after 4 weeks. Serum samples were collected at four and eight weeks after initial immunization, inactivated at 56°C for 30 min and stored at -80°C until use.

Isolation of Antigen-specific B cells in germinal center (B_{GC})

Single-cell suspensions were obtained from the lymph nodes of immunized mice. The cells from each group of mice were pooled together. Antibodies used for staining were anti-mouse GL-7-FITC, CD138-PE, CD38-PE/Cy7, CD93-APC, B220-BV421 and IgD-BV510. All antibodies were used according to manufactures' instructions. To stain the SARS-CoV-2-binding B cells, a mixture of RBD and S proteins were used as baits by biotinylation via Biotin-Protein Ligase/BirA Enzyme (GeneCopoeiaTM). BV711-Streptavidin was used to probe the biotinylated SARS-CoV-2 RBD and S proteins. Antigen-specific B_{GC} gated as GL-7^{+}, $B220^{high}$, $CD38^{low}$, IgD^{-}, $CD93^{-}$, $CD138^{-}$ and SARS-CoV-2 RBD or S^{+} were sorted by BD FACSAria II flow cytometer (BD Biosciences). At last, approximately 9000 (RBD group) and 5000 (S group) SARS-CoV-2 specific cells were sorted by flow cytometry and subjected to the next 10X Genomics for high-throughput single-cell V(D)J sequencing.

Single-cell library construction and sequencing

Sample processing for single B cell receptor (BCR) V(D)J clonotype was done using Chromium Single Cell 5′ Library and the Gel Bead Kit following the manufacturer's user guide (10x Genomics, Pleasanton, CA, CG000086_SingleCellVDJReagentKitsUserGuide_R-evB). After FACS sorting, cells were spun down, resuspended in 3% fetal bovine serum (Sigma-Aldrich, St. Louis, MO)/phosphate buffer solution (Thermo Fisher Scientific, Waltham, MA) and subjected to cell quality control using cell counter. All the processed B cells had cell viability >70%. After determining cell density, cells were injected into three channels, aiming to achieve ~1000-3000 cells per channel. Gel Beads-in-Emulsion (GEMs) were formed in channels of a chip in the 10x Chromium instrument, and then collected into an Eppendorf plate for GEM reverse transcription (GEM-RT) reaction. After GEM clean up, GEM-RT products were subjected to two rounds of nine PCR cycles and 15 PCR cycles using custom primers, followed by SPRIselect (Beckman Coulter, Brea, CA) beads clean up. Single-cell BCR V(D)J Libraries were prepared following the manufacturer's user guide (10x Genomics, Pleasanton, CA, CG000086_SingleCellVDJReagentKitsUserGuide_RevB), and profiled using the Bioanalyzer High Sensitivity DNA kit (Agilent Technologies, Santa Clara, CA) and quantified with kapa Library Quantification Kit (Kapa Biosystems, Wilmington, MA). Libraries were sequenced by paired-end sequencing (2 × 150 bp) on an Illumina NovaSeq (Illumina, San Diego, CA). BCL data were converted to demultiplexed FASTQ files using Illumina bcl2fastq 2.20.

Cell ranger V(D)J Data processing

We retained the first 26 bases for read one covering the 16 nt cell barcode and 10 nt unique molecular identifier (UMI). The FASTQ files were analyzed subsequently. The Cell Ranger Single-Cell Software Suite (versions 3.1.0) was used to perform barcode processing and single-

cell V(D)J analysis (http://10xgenomics.com/).The FASTQ files were processed using the Cell Ranger V(D)J pipeline. Firstly, reads were filtered for valid cell barcodes and UMIs. Cell barcodes with 1-Hamming-distance from a list of known barcodes were considered. A UMI with 1-Hamming-distance from another UMI with more reads for a same barcode was corrected to this UMI with more reads. Then the filtered reads were used to assembly contigs by alignment with the GRCm38 V(D)J reference genome, then defined alignments of V, D and J segments to a contig, identify CDR3 sequences, and from these data determine if a contig is productive, meaning that it is likely to correspond to a functional B cell receptor. Finally, the barcodes were identified as target cells, if they satisfy the following three requirements: 1) There must be a productive, confident contig, and if there is only one such contig, there must be more than one UMI supporting its junction region. 2) There must be at least three filtered UMIs having at least two read pairs each. 3) Compute the N50 value of the number of read pairs per UMI, across all barcodes. If for a given barcode, the maximum read pair count across filtered UMIs is less than 3% of this N50, do not call the barcode a cell. Collection of cells that share a set of productive CDR3 sequences by exact nucleotide match were defined as clonotype. The V(D)J sequences and clonotypes from single cell 5′ data produced by 10 X Chromium Platform were analyzed, searched and visualized by LoupeTM V(D)J Browser v3.0.0. The sequences were further annotated and analyzed using IgBLAST v1.6.1 to identify variable region gene segments and somatic mutations.

Enzyme-linked immunosorbent assay (ELISA)

ELISA plates (Corning) were coated overnight with 2 μg/ml of RBD (the monomer version) or S ectodomain (the 6P version) of SARS-CoV-2 wildtype strain or South Africa strain in 0.05 M carbonate-bicarbonate buffer, pH 9.6, and blocked in 5% skim milk in PBST at 37°C for one

hour. Serum samples were twofold serially diluted and added to each well, incubated at 37ºC for one hour, prior to three further washes and subsequent one hour incubation with goat anti-mouse IgG-HRP (Yeasen) at 37℃. After five washes with PBST, plates were incubated with TMB substrate (Beyotime). Reactions were stopped with 2 M hydrochloric acid, and the absorbance was measured at 450 nm using a microplate reader (PerkinElmer, USA). The endpoint titers were defined as the highest reciprocal dilution of serum to give an absorbance greater than 2.1-fold of the background values. Antibody titer below the limit of detection was determined as half the limit of detection.

Live SARS-CoV-2 neutralization assay

For serum samples, the microcyto pathogenic effect assay was used to determine 50% neutralization titer. Two repeats were set for each serum. 50 μL twofold serially diluted sera were mixed with 50 μL 100 $TCID_{50}$ virus per well in 96-well plates, subsequently incubated in 5% CO_2 incubators for two hours at 37ºC. Both virus and sera were diluted in DMEM with 5% inactivated FBS. Vero E6 cells were resuspended in DMEM with 10% FBS in density 1.5×10^5/mL, 100 μl cells per well were then added to the mixtures of sera and virus. The final concentrations of FBS were 7.5%. After incubating for three days, the CPE of each well was recorded under microscope, and the neutralizing titer was calculated with Karber method, the dilution of 50% protective condition was defined as the neutralizing titer.

For antibodies, each sample was diluted to desired concentration and mixed with an equal volume 100 $TCID_{50}$ virus. The final concentration of antibodies was <0.1 μg/mL, 0.1-0.6 μg/mL, 1-6 μg/mL or >6 μg/mL. Four replicates of each sample were performed. Antibody neutralizing potency was roughly determined as the lowest concentration range which can afford a ≥50% inhibition of live SARS-CoV-2 virus infections.

Protein expression and purification

To facilitate further purification processes, a hexa-His tag was added to the C terminus of signal peptide-RBD (the monomer version) or -S ectodomain (the 6P version). The optimized RBD or S gene was cloned into the pCAGGS vector (Beijing BioMed Gene Technology) with *Eco*R I and *Xho* I (Thermo Fisher Scientific) restriction sites, resulting in a pCAGGS-sp-RBD-His or pCAGGS-sp-S-His plasmid. 293T cells were seeded in 15 cm dishes at 5,000,000 cells/dish. Eighteen hours later, the cells were transfected with pCAGGS-sp-RBD-His or pCAGGS-sp-S-His plasmid by using PEI Transfection Reagent (Alfa). Four to six hours later, the medium was removed and cells were washed with PBS for three times, followed by addition of Dulbecco's Modified Eagle medium (DMEM) Medium. The supernatant was collected after three and seven days. The collected supernatant was centrifuged at 5,000 g for 10 minutes before filtration using 0.22 μm Membrane Filter (Millipore), and soluble protein was purified by Ni affinity chromatography using a HisTrap HP 5-ml column (GE Healthcare). The sample was further purified via gel filtration chromatography with a Superdex 200 column (GE Healthcare) in a buffer composed of 20 mM Tris-HCl (pH 8.0) and 150 mM NaCl.

Expression and quantification of monoclonal antibodies (mAbs)

The variable regions of antibodies were linked with the coding sequences for mouse IgG2a constant region to generate full-length mAbs. Small productions of mAbs were performed by co-transfection of paired full-length H chain and L chain genes into 293T cells precoated in 6-well plate. The culture supernatant was collected after three days. Quantification of mAbs in supernatant was performed with a commercial Mouse IgG2a ELISA Kit (Multi Sciences) according to the manufacturer's instructions. The assay is based on a double-antibody sandwich principle that detects mouse IgG2a in samples. Briefly, a monoclonal antibody specific for

mouse IgG2a was pre-coated onto plate wells. Standards or antibody samples were added to the wells and incubated for two hours at room temperature. After six washes, plates were incubated with HRP-conjugated detected antibody for one hour at room temperature, followed by six washes and incubation with TMB substrate. The absorbance at 450 nm was measured. A standard curve of absorbance at 450 nm versus concentration was fit with a linear equation for accurate mouse IgG2a quantification.

Biolayer Interferometry assay (BLI)

Both the real-time binding and blocking experiments were performed by biolayer interferometry using an Octet RED96 biosensor (Pall ForteBio) at 25°C in PBST buffer with the plate shaking at the speed of 1,000 rpm. Briefly, AMC biosensors from ForteBio were used to capture antibodies in cell culture onto the surface of the AMC biosensor within 400s and then reached baseline. For binding experiments, sensors were subjected to the association step containing 400 nM purified antigens for 300s and then dissociated for 300s. Antibodies from the RBD group were subjected to purified RBD protein, and antibodies from the S group were subjected to S ectodomain, RBD, NTD, S1 and post-fusion S2 protein. For blocking experiments, sensors were subjected to the association step containing 800 nM purified RBD protein for 300 s and then went to baseline followed by association with 1uM purified ACE2 protein for 300 s. Data was analyzed by FortéBio Data Analysis.

Mutant pseudovirus neutralizing assay

Using mutant pseudovirus neutralizing assay, we determined neutralizing activity of vaccinated mouse sera against various SARS-CoV-2 pseudoviruses that harbored key mutations in S protein. Briefly, 100 µl serial dilution of vaccinated mouse sera or monoclonal antibody preparations were added into 96-well plates. After that, 50 µl pseudoviruses with concentration

of 1300 TCID50/ml were added into the plates, followed by incubation at 37°C for 1h. Afterward, Huh-7 cells were added into the plates (2×10^4 cells/100 mL cells per well), followed by incubation at 37°C in a humidified atmosphere with 5% CO_2. Chemiluminescence detection was performed after 24 hours incubation. The Reed-Muench method was used to calculate the virus neutralization titer. The results are based on 3-5 replicates unless specified.

FACS assay

The activity of mAbs to block the binding between the SARS-CoV-2 RBD and hACE2 was assessed by FACS. HEK293T cells were transiently transfected with the pEGFP-N1-hACE2 expression plasmid for 48 h. The SARS-CoV-2 RBD protein at a concentration of 200 ng/ml was mixed with the mAbs at a molar ratio of 1:10 and incubated at 4°C for 1 h. Then mixtures were added to 3×10^5 HEK293T cells expressing hACE2 and incubated at room temperature for 30 minutes. After washing with PBS three times, the cells were stained with anti-His APC-conjugated antibody for another 30 min and analysed using flow cytometry (BD FACSCalibur).

Competition binding analysis

Detecting antibodies (CB6, B38, P2B-2F6, CovA2-39, S309, REGN10987, EY6A and S2A4) were conjugated with biotin using EZ-Link™ Sulfo-NHS-Biotin (Thermo Scientific) following the manufacturer's protocol. Elisa plates (Corning) were coated with 2 μg/mL SARS-CoV-2 RBD protein overnight at 4°C and blocked in 5% skim milk in PBST at 37ºC for 1 hour. Detecting antibodies at the final concentration of 1 μg/mL and blocking antibodies at the final concentration of 200 μg/mL were added to the same well and incubated for 1 h at 37°C. After washing with PBST, streptavidin conjugated with HRP (Yeasen) was added and incubated for 1 h at 37°C. After five washes with PBST, plates were incubated with TMB substrate (Beyotime). Reactions were stopped with 2 M hydrochloric acid. The competition value was determined by

comparing the binding value of detecting antibody in the presence of blocking antibody divided by the binding value of detecting antibody alone.

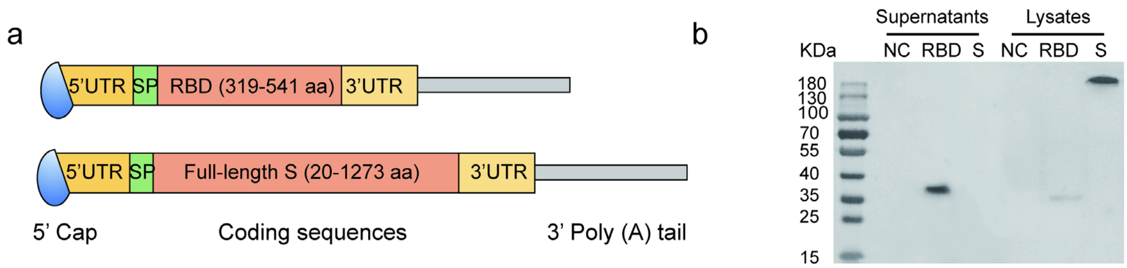

Fig. S1. Construction and *in vitro* expression of SARS-CoV-2 RBD- and S-based mRNA vaccine. (**a**) Schematic of the mRNA vaccine design. The SARS-CoV-2 mRNA vaccines encode the signal peptide (SP), receptor-binding domain (RBD) or full-length spike (S, the 6P variant) protein from SARS-CoV-2 strain Wuhan/IVDC-HB-01/2019. (**b**) RBD- and S-encoding mRNA were transfected into HEK293T cells. Antigen expression in the cell lysate and supernatant was analyzed by Western blotting.

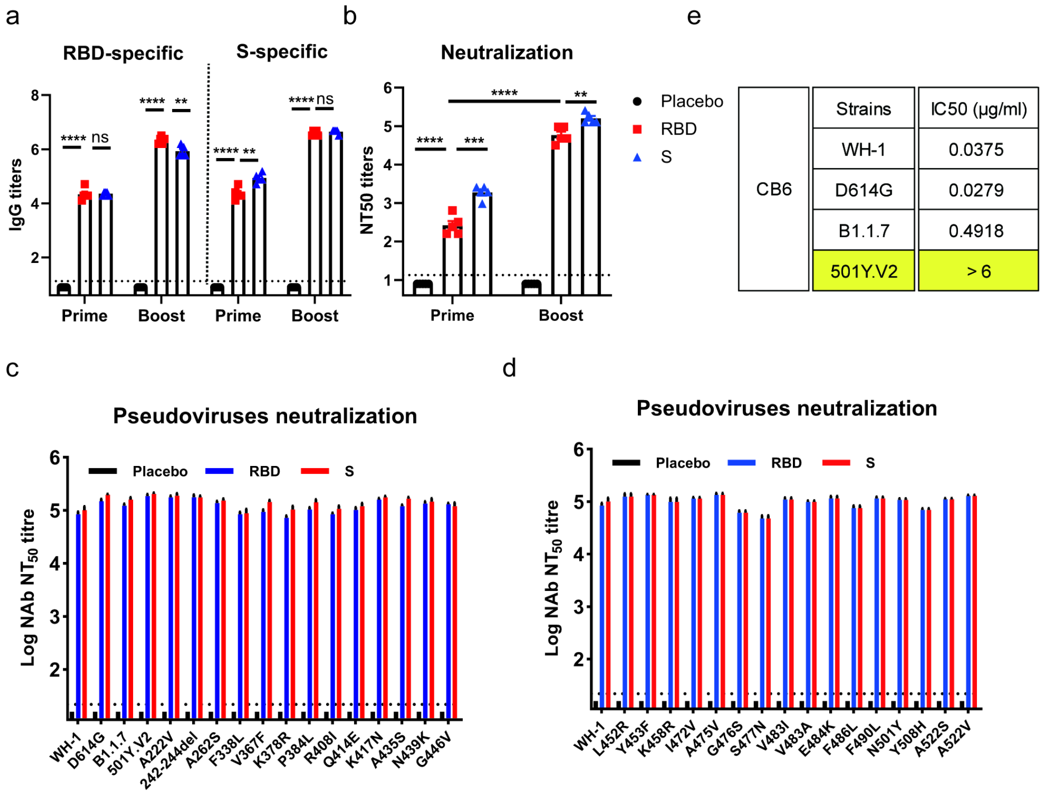

Fig. S2. Immunogenicity of SARS-CoV-2 RBD- and S-based mRNA vaccine. Groups of 6-8-week-old female BALB/c mice (n=5 or 6) were immunized intramuscularly (i.m.) with two doses of SARS-CoV-2 RBD or S mRNA vaccine or with a placebo at a four-week internal. Sera at four and eight weeks post prime immunization were collected. (**a**-**b**) SARS-CoV-2 RBD (monomer) and S (the 6P version) specific IgG and neutralizing antibody titers against live virus were determined as shown in (**a**) and (**b**), respectively. (**c**-**e**) Neutralizing activity of vaccinated mouse sera and CB6 (control antibody) against various SARS-CoV-2 mutant pseudovirus. Serial dilutions of RBD- and S-elicited sera collected at eight weeks post prime vaccination or CB6 were individually mixed with pseudovirus that harbored key mutations in S protein at 37°C for 1h before added to Huh-7 cells for incubation of 24 h to determine the NT_{50} for sera or IC_{50} for CB6. (**c**-**d**) Neutralizing NT50 titers of elicited mouse sera by RBD and S mRNA vaccinations against various mutant pseudovirus. (**e**) Neutralizing activity of the CB6 control against original

and mutant pseudovirus. Data are means ± SEM (standard error of the mean). Comparison was performed by Student’s t-test (unpaired, two-tailed). Data are one representative result of two independent experiments.

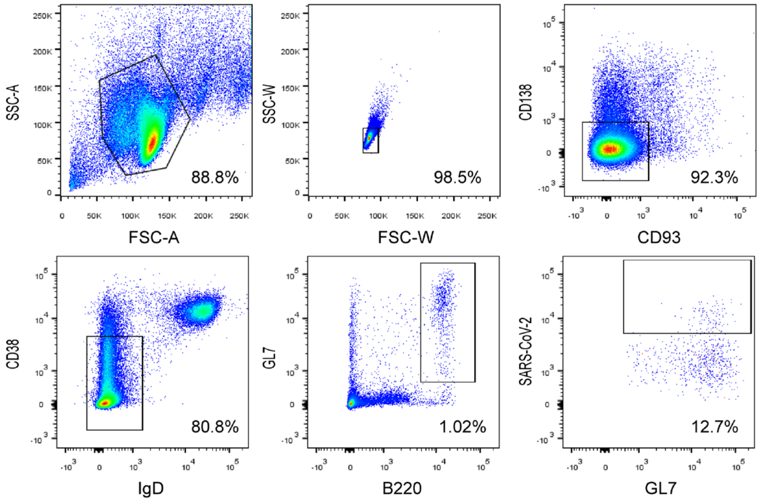

Fig. S3. FACS plots showing the gating strategies to isolate single SARS-CoV-2 specific B_{GC}. The SARS-CoV-2 specific B_{GC} (GL-7+ B220hi CD38low IgD- CD93- CD138-) from the lymph nodes of a group of BALB/c vaccinated with RBD and S vaccines were sorted by flow cytometry.

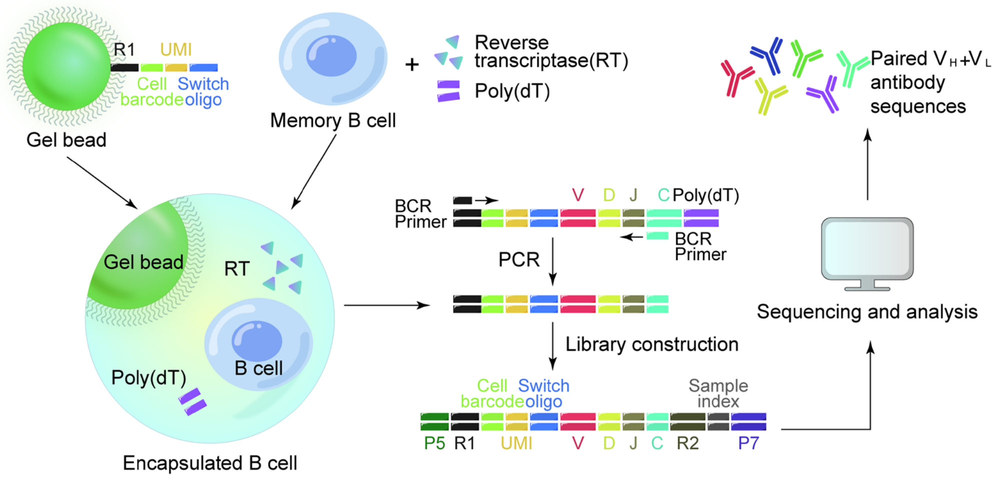

Fig. S4. Schematic representation of single cell immune profiling solution to obtain paired and full-length V(D)J sequences of Immunoglobulin (Ig) by 10X Genomics.

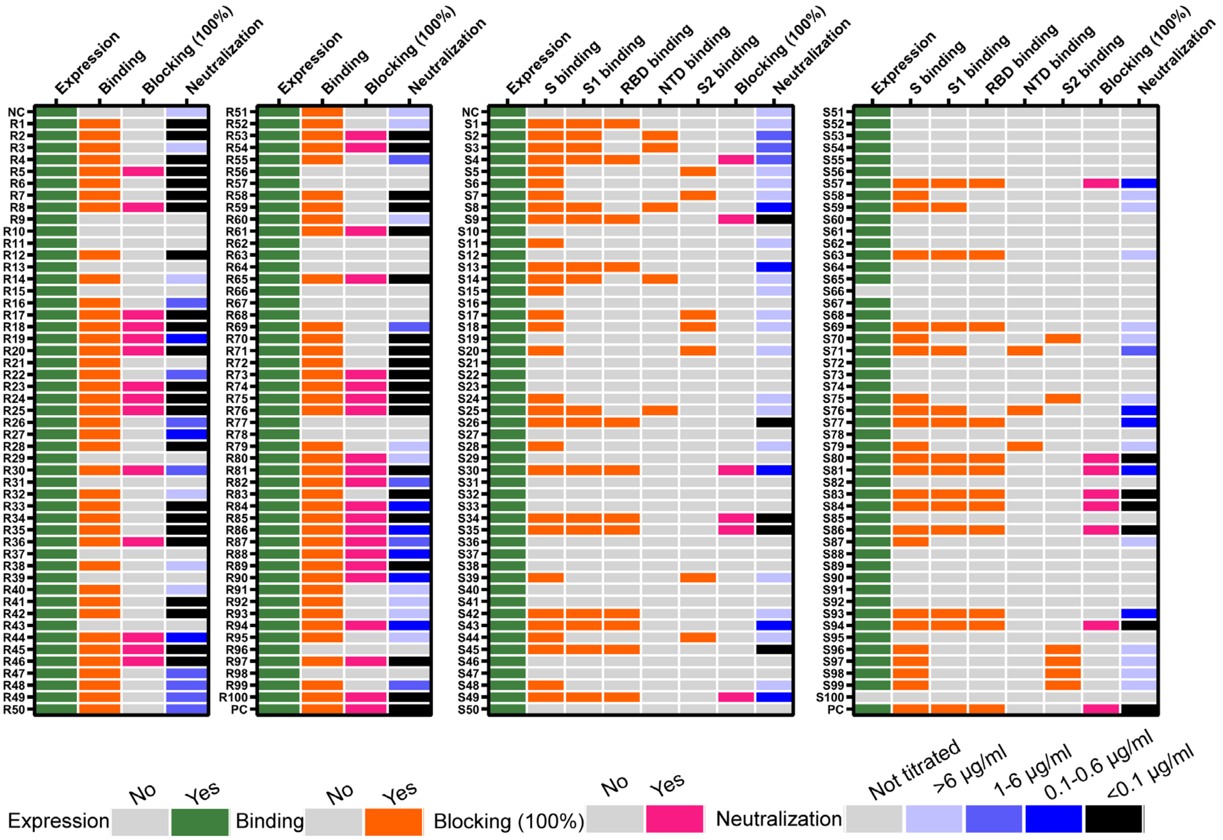

Fig. S5. Antibody characteristics of the recovered top 100 frequent mAbs in each antibody repertoire elicited by SARS-CoV-2 RBD and S vaccinations. Expression, epitope mapping, blocking and neutralization of all mAbs expressed in this study. The VH and VL sequences of 200 mAbs were codon-optimized and synthesized, and each VH and VL gene were then cloned into mouse IgG2a and Igκ expression vectors, respectively. The mAbs derived from the RBD and S groups were termed as R1-100 and S1-100, respectively, and were expressed by co-transfection of paired full-length H chain and L chain genes into 293T cells. Antibody expression in supernatant was detected by a commercial IgG2a quantity kit. Epitope binding and complete blocking of RBD to hACE2 of mAbs were assayed by Octet. Authentic SARS-CoV-2 neutralizing activities of mAbs were determined by a CPE-based microneutralization assay.

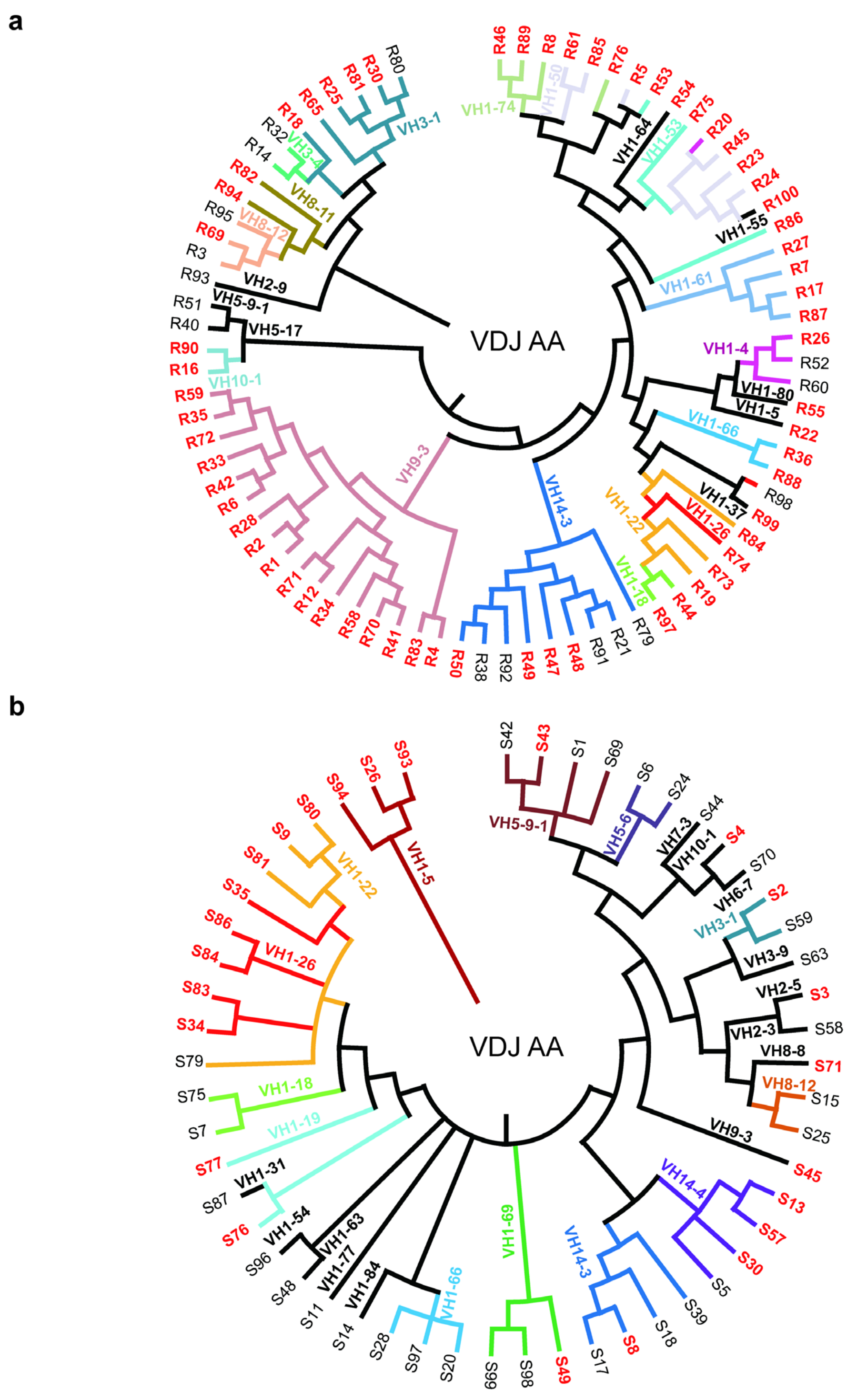

Fig. S6. Phylogenetic trees of RBD- or S-binding mAbs synthesized in this study. The phylogenetic trees of the RBD- or S-specific mAbs were reconstructed based on the VH amino

acid sequences by using a neighbor-joining method in MEGA11 software. (**a**) Phylogenetic tree of synthesized RBD-reactive mAbs from the RBD-elicited antibody repertoire. (**b**) Phylogenetic tree of synthesized S-reactive mAbs from the S-elicited antibody repertoire. The mAbs exhibiting SARS-CoV-2 neutralizing activity were indicated as bold red, whereas the non-neutralizing mAbs was written in regular.

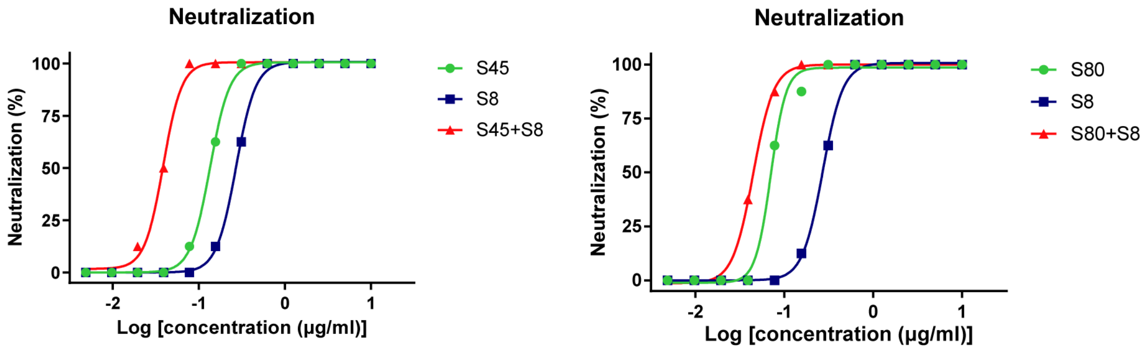

Fig. S7. Synergistic neutralization of authentic SARS-CoV-2 virus by NTD- and RBD-directed mAbs from the S group. S8 (an NTD-directed mAb) was mixed with S45 or S80 (RBD-directed mAbs) from the S group at a weight ratio of 1:1. Neutralizing activities of S8, S45, S80 and antibody mixtures against authentic SARS-CoV-2 virus were assayed by a CPE-based microneutralization assay. Experiments were performed twice, and one set of representative detail is displayed.

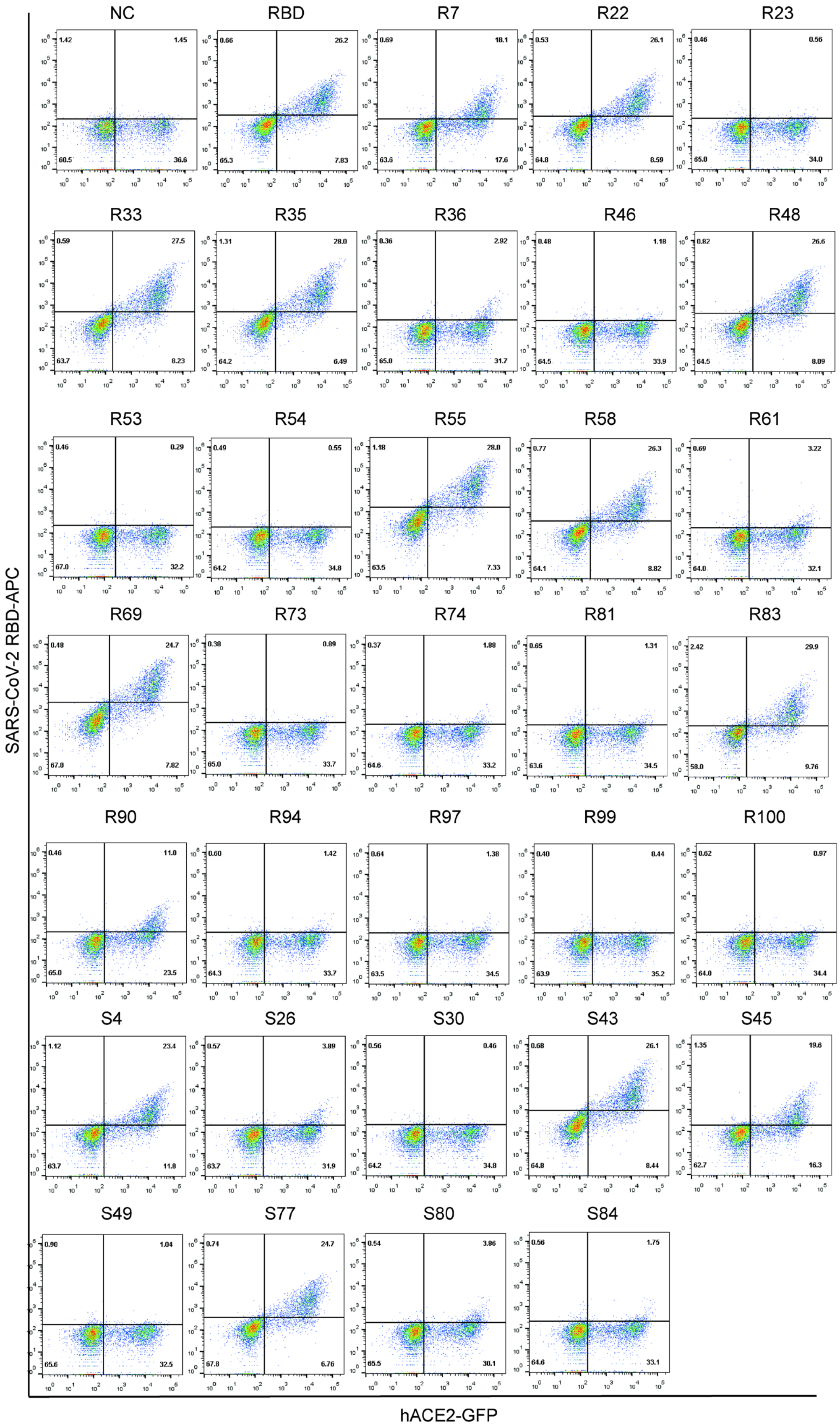
NC
RBD
R7
R22
R23
R33
R35
R36
R46
R48
R53
R54
R55
R58
R61
R69
R73
R74
R81
R83
R90
R94
R97
R99
R100
S4
S26
S30
S43
S45
S49
S77
S80
S84
SARS-CoV-2 RBD-APC
hACE2-GFP

Fig. S8. Ability testing of mAbs from the RBD- and S-elicited antibody repertoires for blocking hACE2 binding to RBD in a FACS-based assay. hACE2-GFP fusion protein was transiently expressed on the surface of HEK293T cells, and stained with SARS-CoV-2 RBD protein pre-incubated with tested mAbs. Experiments were performed twice, and one set of representative detail is displayed.

Detecting antibody

Blocking antibody

		Class 1		Class 2		Class 3		Class 4	
mAb	VH germline	CB6	B38	CovA2-39	P2B-2F6	S309	10987	EY6A	S2A4
R7	VH1-61	103.14%	81.88%	99.83%	14.58%	74.60%	37.41%	111.43%	95.70%
R22	VH1-5	99.46%	106.72%	92.93%	27.17%	93.40%	87.41%	115.33%	103.51%
R23	VH1-4	7.42%	55.91%	13.14%	94.60%	107.61%	95.60%	90.50%	94.90%
R33	VH9-3	104.01%	71.07%	108.99%	12.08%	56.84%	9.39%	103.21%	91.03%
R35	VH9-3	103.14%	74.16%	98.74%	12.96%	56.07%	13.32%	95.38%	91.62%
R36	VH1-66	112.82%	79.57%	92.90%	13.61%	52.80%	20.95%	92.40%	97.57%
R46	VH1-74	27.15%	65.95%	44.63%	97.33%	111.08%	97.90%	93.86%	94.19%
R48	VH14-3	109.43%	100.66%	111.32%	88.36%	121.43%	103.91%	95.35%	89.02%
R53	VH1-53	7.99%	72.11%	10.96%	226.28%	87.70%	100.16%	94.06%	100.26%
R54	VH1-64	6.77%	53.31%	7.22%	181.54%	92.77%	102.99%	101.23%	106.34%
R55	VH1-80	98.40%	98.16%	105.11%	92.72%	108.73%	83.37%	63.17%	61.13%
R58	VH9-3	114.59%	72.09%	106.41%	11.25%	54.84%	8.37%	89.50%	103.32%
R61	VH1-50	24.26%	32.37%	26.86%	29.72%	36.41%	40.43%	96.97%	97.04%
R69	VH8-12	99.63%	84.16%	85.76%	100.51%	124.33%	102.52%	92.67%	85.64%
R73	VH1-22	27.32%	74.90%	63.06%	94.68%	95.82%	107.66%	102.98%	96.07%
R74	VH1-26	14.50%	62.45%	23.82%	92.32%	103.78%	64.43%	104.67%	99.99%
R81	VH3-1	11.62%	15.53%	6.38%	15.25%	24.42%	6.38%	99.41%	101.72%
R83	VH9-3	100.30%	67.61%	99.36%	12.50%	55.50%	14.48%	96.88%	100.43%
R90	VH10-1	63.48%	76.38%	91.60%	22.84%	107.22%	90.41%	103.86%	102.16%
R94	VH8-11	106.20%	53.68%	103.16%	92.16%	97.37%	28.35%	82.37%	33.05%
R97	VH1-18	8.22%	78.07%	10.74%	176.75%	88.96%	99.93%	95.82%	100.62%
R99	VH1-37	105.07%	68.61%	99.57%	92.08%	108.09%	28.31%	31.25%	12.28%
R100	VH1-55	26.30%	76.57%	81.27%	38.59%	106.51%	67.94%	89.95%	92.06%
S4	VH10-1	98.75%	99.59%	83.06%	80.26%	109.03%	96.48%	97.35%	92.09%
S26	VH1-5	90.13%	19.05%	105.97%	12.54%	80.24%	11.02%	96.16%	88.17%
S30	VH14-4	94.97%	41.08%	104.14%	13.30%	54.79%	17.99%	26.39%	23.07%
S43	VH5-9-1	108.61%	95.65%	107.85%	19.17%	64.81%	56.69%	98.51%	109.15%
S45	VH9-3	115.50%	85.14%	96.43%	12.91%	58.31%	11.62%	102.18%	93.30%
S49	VH1-69	91.89%	82.53%	96.12%	90.44%	94.82%	23.42%	62.20%	17.33%
S77	VH1-19	102.46%	108.91%	100.61%	91.86%	113.17%	105.19%	100.49%	93.25%
S80	VH1-22	9.71%	53.65%	14.47%	92.83%	107.96%	100.82%	86.60%	94.12%
S84	VH1-26	14.56%	64.43%	28.45%	100.37%	106.22%	111.50%	95.75%	95.04%

Fig. S9. Competition results of representative RBD-reactive mAbs against reference mAbs. Numbers in the box indicate the percentage binding of reference detecting mAbs in the presence of assayed blocking mAbs compared with the binding of reference detecting mAbs in the absence of assayed blocking mAbs. Data are one representative result of two independent experiments.

Table S1 Frequencies of the V gene usage

IGHV				IGLV			
RBD	Frequency (%)	S	Frequency (%)	RBD	Frequency (%)	S	Frequency (%)
IGHV9-3	15.55	IGHV3-1	7.64	IGKV5-45	13.48	IGKV8-30	7.36
IGHV14-3	8.24	IGHV14-3	6.45	IGKV8-30	13.43	IGKV13-84	6.24
IGHV3-1	6.09	IGHV1-9	5.32	IGKV12-44	7.61	IGKV6-32	4.75
IGHV1-74	5.67	IGHV5-6	4.92	IGKV3-4	5.21	IGKV1-135	4.33
IGHV1-61	4.70	IGHV5-9-1	4.85	IGKV4-55	4.68	IGKV6-23	4.04
IGHV1-50	4.40	IGHV14-4	4.58	IGKV3-2	4.61	IGKV9-120	4.04
IGHV8-12	3.10	IGHV1-77	4.05	IGKV6-15	4.18	IGKV1-110	3.68
IGHV1-18	2.54	IGHV9-3	3.79	IGKV1-110	3.77	IGKV14-111	3.44
IGHV10-1	2.49	IGHV1-66	3.59	IGKV3-5	2.88	IGKV3-2	3.38
IGHV1-4	2.38	IGHV1-18	3.32	IGKV1-135	2.74	IGKV9-124	3.38
IGHV1-9	2.38	IGHV1-69	3.06	IGKV9-124	2.33	IGKV4-61	2.91
IGHV5-4	2.38	IGHV7-3	2.99	IGKV17-127	2.28	IGKV6-20	2.79
IGHV1-53	2.21	IGHV1-22	2.46	IGKV4-68	2.26	IGKV4-59	2.67
IGHV1-66	2.13	IGHV1-34	2.26	IGKV3-12	1.87	IGKV1-117	2.38
IGHV3-4	2.05	IGHV8-12	2.19	IGKV4-50	1.75	IGKV12-41	2.38
IGHV4-1	2.02	IGHV3-6	2.13	IGKV4-72	1.68	IGKV3-5	2.32
IGHV5-17	2.02	IGHV5-4	1.93	IGKV14-111	1.63	IGKV5-39	2.20
IGHV1-22	1.91	IGHV14-1	1.86	IGKV12-98	1.37	IGKV6-17	2.20
IGHV5-12	1.69	IGHV1-5	1.79	IGKV3-1	1.32	IGKV8-24	2.14
IGHV1-26	1.55	IGHV10-1	1.79	IGKV4-59	1.30	IGKV8-19	2.08
IGHV1-69	1.55	IGHV1-26	1.73	IGKV10-96	1.23	IGKV12-46	2.02
IGHV5-9-1	1.52	IGHV1-71	1.73	IGKV13-85	1.18	IGKV4-55	1.84
IGHV5-6	1.49	IGHV2-2	1.66	IGKV6-17	1.13	IGKV10-96	1.54
IGHV6-6	1.27	IGHV2-9	1.66	IGKV3-10	1.08	IGKV4-50	1.48
IGHV1-5	1.22	IGHV2-3	1.59	IGKV6-32	0.98	IGKV3-7	1.37
IGHV1-19	1.16	IGHV2-5	1.59	IGKV12-46	0.91	IGKV3-9	1.37
IGHV1-37	1.02	IGHV1-19	1.40	IGKV9-123	0.89	IGKV6-15	1.37
IGHV2-9	0.86	IGHV1-84	1.33	IGKV1-117	0.72	IGKV19-93	1.37
IGHV14-2	0.80	IGHV1-54	1.13	IGKV2-137	0.60	IGKV12-44	1.31
IGHV8-8	0.75	IGHV6-6	1.13	IGKV3-7	0.58	IGKV3-12	1.07
IGHV1-64	0.72	IGHV2-6	1.06	IGKV4-74	0.55	IGKV3-3	1.01
IGHV2-6	0.72	IGHV4-1	1.00	IGKV5-39	0.53	IGKV12-98	1.01
IGHV8-11	0.69	IGHV1-4	0.93	IGKV13-84	0.53	IGKV4-79	0.95
IGHV3-6	0.66	IGHV5-17	0.93	IGKV5-43	0.48	IGKV6-14	0.89
IGHV1-52	0.64	IGHV1-53	0.80	IGKV9-120	0.48	IGKV2-137	0.83
IGHV14-1	0.64	IGHV5-12	0.73	IGKV4-80	0.46	IGKV3-4	0.83
IGHV2-6-8	0.61	IGHV9-1	0.73	IGKV6-23	0.43	IGKV3-10	0.71
IGHV1-42	0.55	IGHV1-15	0.60	IGKV5-48	0.41	IGKV4-68	0.71
IGHV2-5	0.55	IGHV1-82	0.53	IGKV12-89	0.41	IGKV17-127	0.71
IGHV5-9	0.55	IGHV8-8	0.53	IGKV8-19	0.38	IGKV3-1	0.59
Others (<0.5)	6.53	Others (<0.5)	6.25	IGKV12-41	0.38	IGKV6-25	0.59
				IGKV8-24	0.36	IGKV8-28	0.59
				IGKV10-94	0.36	IGKV4-91	0.53
				IGKV6-25	0.34	IGKV2-109	0.48
				IGKV14-100	0.34	IGKV8-21	0.48
				IGKV4-63	0.29	IGKV16-104	0.48
				IGKV16-104	0.29	IGKV4-72	0.42
				IGKV4-61	0.24	IGKV5-43	0.42
				Others (<0.2)	3.05	IGKV5-48	0.42
						IGKV4-53	0.36
						IGKV15-103	0.36
						IGKV4-74	0.30
						IGKV8-27	0.30
						IGKV10-94	0.30
						IGKV4-57	0.24
						IGKV5-45	0.24
						Others (<0.2)	1.84
